# Trajectories of depressive symptoms in early to mid-adolescence: associations with school pedagogical and social climate

**DOI:** 10.1177/14034948241277048

**Published:** 2024-10-24

**Authors:** Cristian Bortes

**Affiliations:** 1 Department of Social Work, Umeå University, Sweden; 2 Department of Global Public Health, Karolinska Institutet, Sweden

**Keywords:** depressive symptoms, mental health, adolescence, school climate, educational environment, latent class growth analysis

## Abstract

**Aims:**

Adolescence is a critical period for mental health development, yet research exploring how contextual factors influence the development of depressive symptoms remains limited. This study explored trajectories of depressive symptoms during early to mid-adolescence and their association with various aspects of school climate.

**Methods:**

The study sample comprised 3671, 7th-grade students (aged 12–13 years) from 101 schools across Sweden, followed longitudinally across three time points spanning grades 7, 8 and 9. Depressive symptom trajectories were identified using latent class growth modelling. The Pedagogical and Social Climate questionnaire assessed school climate, and multinomial logistic regression was employed to predict trajectory membership based on sociodemographic and school climate factors.

**Results:**

Four distinct developmental patterns of depressive symptoms emerged: ‘Sustained low symptoms’ (76.7%), ‘Low–increasing’ (10.9%), ‘Sustained high symptoms’ (7.9%), and ‘High–decreasing’ (4.5%). Gender, parental education and six specific school climate factors, out of the total 19 examined, significantly distinguished these trajectory classes. Positive teacher expectations and strong principal involvement were associated with more favourable trajectories, whereas teaching activities, teacher support and communication between school and home were associated with less favourable trajectories, suggesting a nuanced understanding of their relationship with depressive symptom trajectories.

**Conclusions:**

**Few school factors were found to be relevant to depressive symptoms, highlighting the importance of considering external factors beyond the school environment in supporting adolescents during this developmental stage. Although the findings are multifaceted, it is primarily positive interpersonal relationships, especially through teacher expectations, that stand out as significant factors in promoting youth mental health.**

## Introduction

Research on the development of depressive symptoms during adolescence indicates significant variation in both initial levels and rates of change over time [1–3]. While studies consistently identify four distinct developmental patterns—low and stable levels, high and stable levels, high initial levels that decrease over time and low initial levels that increase over time [1–3]—researchers have primarily focused on individual-level risk and protective factors. Factors such as female gender [4], pubertal timing [5], low socioeconomic status [6], stressful life events [7], minority group membership [8], parental history of psychiatric disorders [9] and problems with parents and peers [10] commonly distinguish adolescents on higher versus lower symptom trajectories, shedding light on individual characteristics affecting depressive symptom manifestation and progression. However, scant attention has been given to how contextual factors, like the school environment, shape these trajectories [1–3], despite schools’ significant roles in adolescents’ lives [11]. This lack of research leaves a gap in understanding and poses a challenge in addressing adolescent mental health needs.

School climate, a multidimensional concept, encapsulates most aspects of the school environment [12,13]. It encompasses perceptions and experiences of school personnel, students, and parents regarding a school’s norms, goals, values, interpersonal relationships, quality of teaching and learning, as well as organizational structures. Previous research has conceptualized school climate across four overarching dimensions: academic, community, safety and institutional environment [12,13]. The academic dimension relates to the educational environment, including curricula, teaching methods, teacher qualifications and professional development. The community dimension, or social environment, refers to the interpersonal relationships within the school, enriched by positive and supportive interactions among staff and students, shared decision-making, school–family collaboration and feelings of school connectedness. The safety dimension entails the physical and emotional security provided by the school, including order and discipline. The institutional environment pertains to organizational and infrastructural characteristics, such as the school’s size, condition of buildings, cleanliness, space and materials.

The current research on school climate has mainly centred on its relationship with students’ academic performance, overlooking a critical aspect: its relationship with mental health [12,13]. While some earlier studies have explored a few dimensions of school climate, such as community and safety and identified links between positive interpersonal relationships and reduced psychopathology [14,15], research on other factors like the academic dimension and broader institutional influences on mental health remains limited. Additionally, existing research often relies solely on single-informant reports, thereby missing out the combined perspectives from students, teachers and parents [16]. Understanding how multiple aspects of the ‘school experience’, as perceived by multiple informants, contribute to youth mental health is crucial for fully grasping the school environment’s role in shaping health development [13].

This study aims to bridge the gap between research on depressive symptom development and research on school climate by examining their relationship. Specifically, it investigates how different aspects of school climate may predict the trajectories of depressive symptoms from early to mid-adolescence. The study offers two main contributions: first, by examining the role of the school environment, it advances the understanding of contextual factors influencing the trajectories of depressive symptoms. Second, it expands the limited existing research on the relationship between school climate and mental health by comprehensively assessing 19 dimensions of school climate using multi-informant ratings.

## Methods

### Data

I use data from the longitudinal KUPOL project [17], which enrolled students from private and public schools across eight counties in southern and mid-Sweden. Initially, 541 schools were contacted, with 101 (19%) agreeing to participate. This resulted in a sample of 12,512 7th-grade students, of whom 3959 (31.5%) consented to participate, with a gender distribution of 51.2% girls. The cohort rests on two sub-samples of adolescents recruited during the school years 2013–2014 and 2014–2015. A flowchart depicting participant recruitment can be found in Fig. A1 of the supplementary materials. Additional details on the cohort profile are available in the referenced sources [16,17].

The baseline measurement for this study included 3671 adolescents in the 7th grade. These adolescents were followed up over the next 2 years, with response rates of 95.4% (3502) in grade 8 and 91.3% (3351) in grade 9. For clarification, in the Swedish educational system, students in the 7th, 8th and 9th grades generally correspond to ages 12–13, 13–14 and 14–15 years, respectively.

Participation was voluntary, and written informed consent was obtained from both the students and their legal guardians. Data were collected from multiple informants: adolescents’ health information from self-reported questionnaires, school-level factors (school climate) from questionnaires completed by schoolteachers and the schools’ 9-grade students, and additional sociodemographic information from parents. The KUPOL study was approved by a Swedish Regional Ethical Review Board (reference numbers 2012/1904-31/1 and 2016/1280-32).

### Variables

#### Depressive symptoms

The 20-item self-report Center for Epidemiological Studies Depression Scale for Children (CES-DC) was used to measure depressive symptoms. Each item asks how often the respondent has experienced a certain symptom in the past week, with responses ranging from 0 (Not at all) to 3 (A lot). Total scores are generated by summing responses (range: 0–60). Scores indicate degrees of depressive symptoms, with 15 indicating risk for depression. The CES-DC is an established and widely used reliable measure of depressive symptoms that has been extensively validated in Sweden and internationally [18,19].

#### School climate

The Pedagogical and Social Climate (PESOC) questionnaire [20,21] was used to measure school climate. The instrument has separate versions for teachers and students, both covering dimensions of school climate such as order, safety and discipline, teaching and learning, social relationships, shared parent and teacher norms and quality of school facilities.

The teacher version (T-PESOC), given for completion to all teaching personnel in the participating schools [20], includes 59 items that assess teachers’ perceptions of the following dimensions of their school: teachers’ expectations for students’ behaviour and academic performance; perceived teacher agreement about school goals, norms, and rules; student focus; basic assumptions about students’ ability to learn; communication between school and home; teacher interaction and cooperation; teachers’ confidence and professional development; teaching activities; evaluation of students’ academic progress; principal’s pedagogical leadership; teachers’ perception of the school management’s involvement and support.

The student version (S-PESOC) was given to all 9-grade students in the schools [21]. Since Swedish compulsory secondary education (*högstadium*) consists of grades 7–9, with 9th grade being the final year, these students had typically spent at least 3 years at the school. Hence, they were most familiar with the school’s ethos, making them the most reliable informants. Thus, when the longitudinal cohort of adolescents, followed from 7th to 9th grade, answered their first questionnaire about health in the 7th grade, a different group of adolescents in the 9th grade had completed the school climate questionnaire. Consequently, the students assessing the school climate were not the same as the participants in the longitudinal sample (except during the final assessment when some overlap occurred).

The student version of the school climate questionnaire, comprising 53 items, evaluates students’ perceptions of the following dimensions of their school: expectations; perceptions of teacher norms; teachers’ support; teaching activities; student participation; school environment; school and home; school management. The questionnaire took between 15 and 45 minutes to complete [21]. The data collection procedure differed slightly for different classes within the same school, mainly due to the need to adapt the process to the schools’ wishes. In the majority of cases, the data collection was supervised by a research assistant, and sometimes a teacher was present.

All items in both versions are in the form of statements with four scoring response options ranging from 1 (Not at all) to 4 (Completely agree) and a ‘Don’t know’ option. Higher scores reflect more favourable school characteristics. Scores for subscales, measuring each dimension, are obtained by averaging the responses, with don’t know responses treated as missing. Both questionnaires have good psychometric properties according to validation studies [20,21] based on the same sample of schools recruited for this study. See supplementary materials for detailed information on the specific items, descriptive statistics and reliability coefficients for the subscales.

#### Sociodemographics

Participating students were asked about their gender (girl/boy). Information on parental country of origin and level of education was self-reported by parents of 7th-grade students then categorized as ‘at least one parent born in Sweden’ (yes/no) and ‘at least one parent with university education’ (no/yes), corresponding to International Standard Classification of Education (ISCED) level 5 or above.

### Data analysis

Latent class growth analysis, conducted in Mplus 8 [22], was employed to identify trajectories of adolescent depressive symptoms [23]. Latent class growth models with different class solutions were tested to identify subgroups of adolescents with distinct patterns of change over time. Given that depressive symptoms were only measured at three timepoints (7th, 8th and 9th grades, coded 0, 1 and 2, respectively), the functional form of individual change was assumed to be linear. The selection of the optimal number of trajectory classes was guided by well-established recommendations [24,25], including the Bayesian Information Criterion (BIC), where lower values indicate better model-data fit; the Voung-Lo-Mendell-Rubin likelihood ratio test (VLMR-LRT), which compares models with *n* − 1 and *n* classes and provides corresponding *p* values; non-significant likelihood ratio tests indicating whether models with *n* classes or *n* − 1 classes offer better data fits; the entropy index, where values closer to 1 signify more accurate classification of participants; and both the interpretability and utility of the trajectories [24,25].

After establishing the trajectory classes, multinomial logistic regression analysis was conducted to predict adolescents’ developmental trajectory based on their sociodemographic characteristics (gender, parental level of education and immigrant background) and school climate dimensions. The school climate predictors used in the analysis were based on assessments made by 9th graders in the participating schools at the inception of the longitudinal cohort (whose depressive symptoms were measured)—i.e. when the participants in the longitudinal cohort began grade 7. Thus, the school climate data reflect the perspectives of older students (and teachers), who assessed the school climate first, and these assessments are then utilized as predictors of trajectory class membership in the younger cohort, who assessed their health. This methodological approach provides temporal sequencing, reinforcing the validity of school climate variables as predictors. Continuous predictors were centred at their respective means, while categorical predictors retained their original scaling.

To account for clustering of data (adolescents clustered in schools), the ‘cluster’ and ‘type = complex’ commands in Mplus were used, with school identification number as the cluster variable, providing robust standard error estimates and unbiased test statistics. Missing data were handled using full information maximum likelihood (FIML) estimation. Seven missing data patterns were identified (see Table A4 in supplementary materials for details), with covariance coverage ranging between 0.77 and 0.90, exceeding the recommended 0.10 threshold to reliably use FIML [26,27]. This means that despite some missing data at one or more time points, participants were included in the analysis if they had sufficient data on class indicators, leveraging FIML to maximize the use of available data and ensure robust model estimation.

## Results

### Subgroups of depressive symptom trajectories

Table I presents fit statistics for latent class growth models with different class solutions. The BIC decreased (indicating better fit) toward a five-class model and the VLMR-LRT indicates that it provides a better fit than a four-class model. However, further inspection of the estimated mean trajectories revealed that the five-class model did not yield a fifth trajectory class substantively distinct from four classes derived from the four-class solution. Moreover, the entropy value was higher for the four-class solution, indicating better classification quality. Therefore, the four-class solution was deemed superior in terms of accuracy and parsimony and was selected as the final model for the next analytical step.

**Table I.** Criteria used to decide on optimal solution for number of latent classes.

	BIC	Entropy	VLMR-LRT*
1-class	73,247.09	–	–
2-class	72,568.62	0.84	0.0000
3-class	72,243.22	0.79	0.0001
**4-class**	**72,077.75**	**0.80**	**0.0049**
5-class	71,958.73	0.78	0.0324
6-class	71,983.49	0.66	0.5000

* *p* value; selected class solution in bold.

BIC, Bayesian Information Criterion; VLMR-LRT, Voung-Lo-Mendell-Rubin likelihood ratio test.

**Figure 1.**
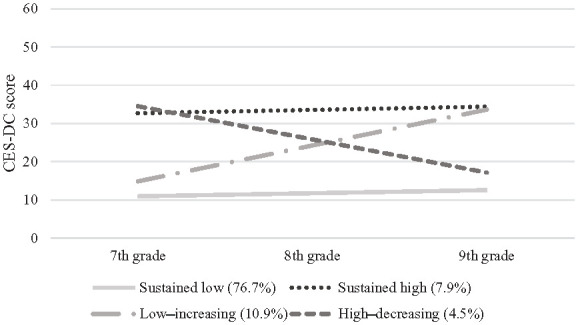
Depressive symptom trajectories from 7th to 9th grade. CES-DC, Center for Epidemiological Studies Depression Scale for Children.

Table II presents growth estimates and class sizes for depressive symptom trajectories. In the four-class model, the largest class (Sustained low symptoms) comprised 76.7% of the sample. Adolescents on this trajectory reported no or mild depressive symptoms in 7th grade, and their symptoms negligibly increased over time. The second class (Low–increasing, 10.9% of the sample) comprised adolescents who reported mild depressive symptoms in 7th grade that increased substantially to significant levels in 9th grade. Adolescents in the third class (Sustained high symptoms, 7.9% of the sample) reported high levels of depressive symptoms in 7th grade that did not significantly change over time. Adolescents in the final class (High–decreasing, 4.5% of the sample) reported high levels of depressive symptoms in 7th grade that substantially decreased over time. (Note: the total number of observations in the final class counts exceeds the number of participants with the most data for a single outcome variable due to the FIML approach, which includes participants with partial data). Figure 1 shows the four trajectories and the estimated mean change.

**Table II.** Growth estimates and class sizes for depressive symptom (CES-DC) trajectories.

Class name	Latent classes		Intercept		Slope	
	*n*	Proportions	Mean	*p*	Mean	*p*
Sustained low	2944	76.7%	10.94	<0.001	0.81	<0.001
Low–increasing	419	10.9%	14.85	<0.001	9.39	<0.001
Sustained high	302	7.9%	32.70	<0.001	0.86	0.376
High–decreasing	172	4.5%	34.53	<0.001	−8.69	<0.001

CES-DC, Center for Epidemiological Studies Depression Scale for Children.

### Sociodemographic and school climate predictors of trajectory membership

Next, the sociodemographic and school climate variables were simultaneously entered as predictors of latent class (trajectory) membership. Specifically, a multinomial logit model was specified to regress the latent class variables on the covariates/predictors. Adolescents in the normative developmental group (the Sustained low class) served as the reference group to identify predictors for adolescents following the High–decreasing, Low–increasing, or Sustained high symptoms trajectories compared with the normative trajectory. The results, including the regression coefficient estimates presented in Table III, are interpreted similarly to a regular multinomial logit model. This means that the coefficients indicate whether specific predictors are either positively or negatively associated with membership in certain trajectory classes *compared with* the reference group (the Sustained low class).

**Table III.** Results from the multinomial logistic regressions predicting class membership.

vs. Class 1 (Sustained low)									
	Class 2 (Low-increasing)			Class 3 (Sustained high)			Class 4 (High-decreasing)		
	Coefficient (SE)	OR	*p*	Coefficient (SE)	OR	*p*	Coefficient (SE)	OR	*p*
Gender (girls/boys)	−2.08 (−13.87)	0.13	**<0.001**	−3.65 (−6.26)	0.03	**<0.001**	−2.08 (−6.07)	0.13	**<0.001**
Parental education	−0.32 (−2.44)	0.73	**0.020**	−0.64 (−2.62)	0.63	**0.009**	−0.08 (−0.43)	0.93	0.666
Immigrant background	−0.54 (−2.22)	0.58	0.054	−0.27 (−0.91)	0.77	0.365	0.47 (1.85)	1.59	0.064
S-PESOC									
Teachers’ expectations	−2.49 (−1.74)	0.08	0.081	−5.24 (−3.17)	0.01	**0.002**	−1.43 (−0.70)	0.24	0.485
Perceptions of teacher norms	1.07 (0.83)	2.93	0.407	−0.53 (−0.29)	0.59	0.769	2.73 (1.82)	15.29	0.068
Teachers’ support	−0.42 (0.48)	0.65	0.626	0.88 (0.50)	2.40	0.621	−3.24 (−2.17)	0.04	**0.030**
Teaching activities	−0.37 (−0.35)	0.69	0.725	1.46 (0.86)	4.32	0.391	−0.53 (−0.38)	0.59	0.704
Student participation	0.35 (0.98)	1.42	0.327	−0.13 (−0.23)	0.88	0.817	−0.21 (−0.39)	0.82	0.694
School environment	0.15 (0.51)	1.16	0.608	0.19 (0.41)	1.21	0.685	0.18 (0.35)	1.20	0.730
School and home	0.57 (1.19)	1.77	0.236	−0.21 (−0.23)	0.81	0.819	1.47 (1.36)	4.34	0.175
School management	−0.51 (−2.38)	0.60	**0.017**	0.25 (0.57)	1.28	0.569	−0.71 (−1.59)	0.49	0.112
T-PESOC									
Teachers’ expectations	0.33 (0.52)	1.39	0.604	1.00 (1.12)	2.71	0.264	1.57 (2.07)	4.81	**0.039**
Perceived teacher agreement	−0.01 (−0.03)	0.33	0.973	0.12 (0.23)	0.51	0.818	−0.28 (−0.63)	2.81	0.531
Student focus	−0.27 (−0.34)	0.77	0.733	–0.04 (–0.03)	0.96	0.978	−0.59 (−0.50)	0.55	0.616
Basic assumptions	0.85 (1.53)	2.34	0.127	–0.76 (–0.69)	0.47	0.488	0.37 (0.35)	1.44	0.726
Communication school–home	1.41 (2.10)	4.10	**0.036**	0.65 (0.60)	1.92	0.548	−1.29 (−1.29)	0.28	0.197
Teacher interaction and cooperation	−0.77 (−1.56)	0.47	0.120	–0.61 (–0.78)	0.54	0.435	−0.10 (−0.12)	0.90	0.906
Teachers’ confidence and professional development	−0.11 (0.18)	1.11	0.860	0.62 (0.52)	1.86	0.605	0.29 (0.25)	1.33	0.804
Teaching activities	1.25 (2.15)	3.51	**0.032**	0.55 (0.38)	1.73	0.708	0.12 (0.13)	1.13	0.893
Evaluation	−0.38 (−1.17)	0.68	0.243	0.08 (0.14)	1.09	0.889	0.07 (0.10)	1.07	0.923
Principal’s pedagogical leadership	−0.01 (−0.03)	0.99	0.973	0.12 (0.23)	1.12	0.818	−0.28 (−0.63)	0.75	0.531
School management’s involvement and support	−0.09 (−0.43)	0.91	0.664	0.24 (0.47)	1.26	0.642	−0.07 (−0.19)	0.93	0.853

Significant associations (*p* < 0.05) marked in bold.

SE, standard error; OR, odds ratio.

Most of the predictors had insignificantly differentiated patterns of associations with the latent trajectory classes, except the sociodemographic char-acteristics, particularly gender. Girls were more likely to follow the Sustained high, Low–increasing or High–decreasing trajectories than the Sustained low symptoms trajectory. This indicates that girls not only belong to the group characterized by consistently high depressive symptoms but also might be particularly sensitive to factors influencing the fluctuation of depressive symptoms during early to mid-adolescence. Additionally, adolescents with at least one parent with a higher education (and presumably commensurate socioeconomic position) appeared to have lower likelihood of following the Low–increasing or Sustained high trajectories compared with the Sustained low symptoms trajectory.

Regarding the school climate predictors, only 6 out of the 19 dimensions exhibit a meaningful pattern of association with the latent trajectory classes. Specifically, the student-rated dimension of School management emerged as a significant predictor, distinguishing the Low–increasing class from the Sustained low class. Recall that school climate predictors are coded so that higher scores, whether from students (S-PESOC) or teachers (T-PESOC), indicate more positive perceptions. Therefore, this finding suggests that favourable student perceptions of their schools’ pedagogical leadership were inversely associated with membership in the Low–increasing class compared with the Sustained low class.

Teacher ratings of Communication between school and home were somewhat counterintuitively associated with the likelihood of membership in the Low–increasing relative to the Sustained low class. Specifically, the results suggest that as teachers rate the school’s communication with parents more positively, the odds increase for students to experience increasing, rather than constantly low, depressive symptoms.

Teachers’ ratings of Teaching activities predicted higher odds of membership of the Low–increasing compared with the Sustained low symptoms class. This suggests that certain elements of what teachers perceive as high-quality teaching, such as encouraging creative thinking, problem-solving skills and effective use of class time (refer to the supplementary materials for detailed items) are associated with an increased likelihood of students experiencing a rise in depressive symptoms over time, rather than consistently low symptoms.

Lastly, students’ ratings of Teachers’ expectations predicted lower odds of membership in the Sustained high class, suggesting that students are less likely to follow a trajectory characterized by constantly high depressive symptoms when teachers have high expectations for their success in school. Similarly, students’ ratings of Teachers’ support, along with teachers’ ratings of Teachers’ expectations, distinguished students in the High–decreasing class from those in the Sustained low class. The latter implies that the prevalence of high depressive symptoms among student cohorts may decrease in schools where school staff hold high expectations for the students.

## Discussion

The findings from this study regarding the trajectories of depressive symptoms among adolescents in Sweden align with the four typical developmental patterns identified in previous research [1–3]. Most of the participating adolescents (76.9%) exhibited either no or minimal depressive symptoms over time (from 7th to 9th grade), while 10.9% experienced low initial levels that increased gradually, 7.9% displayed consistently high symptoms, and the least common pattern (4.9%) demonstrated high initial levels that decreased over time. Additionally, this study adds to existing research by analysing the trajectories of Swedish adolescents, a population not previously examined in research on depressive symptom trajectories, and their associations with previously unexplored school environmental factors [1–3].

The prevalence of depressive symptoms in the KUPOL sample aligns with findings from other studies with more representative samples. Schubert et al. [1] reviewed data from 47 studies on cohorts aged 15 to 25 years, reporting that 5–12% of young people exhibit ‘consistently elevated symptoms’, 1–5% show ‘increasing/decreasing symptoms’ and 60–80% maintain ‘consistently low symptoms’. My findings are consistent with these broader patterns in adolescent mental health research. However, I observed a larger-than-expected ‘low–increasing class’ (10.9%). This can be attributed to the younger age of the participants (13–14 years) at the initial measurements, compared with Schubert et al.’s focus on those aged 15 years and older. Research indicates that adolescents in this younger age group often experience increases in depressive symptoms [2,5], explaining the higher proportion of students in the low–increasing class within the KUPOL sample.

Turning to the school climate predictors, the findings show that out of the 19 dimensions of school climate examined, only six predicted memberships in a trajectory class. This suggests that while schools play important roles in adolescents’ lives [11], only a limited number of factors within the school environment are associated with the trajectories of depressive symptoms. External factors beyond the school setting seem to have a stronger impact on the health trajectories of young people [4–10]. Notably, in this study, sociodemographic variables such as female gender and lower socioeconomic status were more strongly associated with unfavourable trajectories than variables related to the school climate.

Nevertheless, the limited number of predictive factors for trajectory class membership primarily belonged to the academic and community dimensions of the school climate construct. These dimensions encompass elements related to the educational and social environment, respectively [12,13], including aspects of teaching and interpersonal relationships between staff and students.

The dimension that stood out most distinctly was Teachers’ expectations. Student ratings of this dimension were negatively related to odds of membership of the Sustained high class, while teacher ratings were positively related to odds of membership of the High–decreasing class. However, given the low reliability of these subscales (Table A3), it is not entirely clear what specific expectations they measure.

Despite this, these subscales can be understood as capturing general expectations. Thus, it appears important for students to perceive that teachers believe in their abilities and for teachers to collectively hold high expectations for students’ behaviour and academic performance. Educational research has long demonstrated the positive effects of teachers’ expectations on students’ in-school behaviour, such as motivation and academic performance [28]. The findings in this study suggests that these expectations likely have positive effects on outcomes beyond academic performance, specifically on students’ mental health, including depressive symptoms.

Earlier studies have shown that students’ perceptions of elements within the community dimension are positively correlated with their adjustment and negatively correlated with both the prevalence of psychopathology and psychological distress [14,15,29,30]. This study found that another specific element of the community dimension, namely favourable student perceptions of their schools’ management, was associated with a reduced likelihood of depressive symptoms increasing over time. This underscores the potential impact of effective pedagogical leadership on student mental health outcomes.

Additionally, the Teaching activities dimension, as rated by teachers, predicted membership in the Low–increasing class. This suggests that certain aspects of what teachers perceive as good teaching quality may have a negative impact on adolescents’ mental health. It may indicate that certain teaching methods or pedagogical approaches do not foster a positive environment or support students’ emotional well-being. This implies that the way teaching is structured and organized, such as the emphasis on performance-driven activities or the lack of emphasis on emotional support, could inadvertently contribute to the escalation of depressive symptoms over time.

Student-rated Teacher support unexpectedly showed a negative association with the likelihood of students following a trajectory characterized by decreasing depressive symptoms. This contradicts previous research emphasizing the importance of social support for reducing depressive symptoms [14,15,31,32]. However, changing the reference group, this suggests that strong teacher support increases the odds of students following the Sustained low trajectory relative to the High–decreasing trajectory. Therefore, while teacher support may not contribute to reductions in initially high symptoms, it appears beneficial for maintaining symptom stability over time.

Another unexpected finding emerged regarding teacher ratings of Communication between school and home, focusing on parental inclusion and awareness of school affairs, which predicted membership in the Low–increasing class. This suggests that positive perceptions of communication by teachers may not always correlate with improved mental health outcomes for students. However, consistent with the logic applied earlier, i.e. by altering the reference group, this could imply that positive perceptions of communication between home and school may contribute to maintaining symptom stability over time. These unexpected findings deserve to be examined more closely in future research, possibly employing qualitative approaches to explore and understand the contextual and cultural factors at play.

These less straightforward associations partially align with previous indications from KUPOL data, suggesting associations between teacher-rated school climate and heightened risks for students’ poor mental health, particularly internalizing problems [16]. Together, these findings challenge the simplistic notion that seemingly ‘positive’ aspects of school climate directly translate to better mental health outcomes for students, highlighting the complexity of these relationships.

Limitations of the study should be acknowledged. The KUPOL project is based on a non-random sample of schools, with both the school and student samples having a low baseline response rate. Reasons given for not participating included schools already being engaged in other evaluation or research initiatives or undergoing significant organizational changes that constrained their time and resources [20]. Additionally, non-participating schools had a higher proportion of students with foreign-born parents than participating schools [21]. Consequently, standard caveats about generalizing the findings apply, and it is important to consider the potential implications of these limitations.

First, the selection of participating schools may have limited the diversity of school characteristics in the sample, resulting in a narrower range of school climates. Although the KUPOL data include considerable variation among schools in terms of size, location, student-to-teacher ratios and diverse sociodemographic and academic indicators [20,21], selection could still affect the generalizability of the findings.

Second, the sample’s limited representativeness of adolescents suggests caution when extending the results to the broader population of Swedish youth. The under-representation of certain demographic groups may restrict the study’s ability to fully capture the diversity of experiences and needs among adolescents in Sweden. This under-representation could lead to incomplete findings regarding the relationships between school climate and depressive symptoms.

It is also important to acknowledge the role of school-related stress for the deteriorating mental health of youth, particularly within the Swedish context [33–35]. Different schools may cultivate distinct performance cultures, potentially influencing students’ perceptions of academic demands and their experiences of stress. However, the PESOC questionnaire lacks direct measures to assess students perceived academic pressure, leaving a gap in understanding the links between academic stress and mental health *trajectories*.

In addition to school-related stress, there are other confounding variables for depressive symptoms that were not controlled for in this study. Recognizing these limitations highlights the need for future research to gain a more comprehensive understanding of the determinants of depressive symptoms. Since gender emerged as a significant predictor of trajectory class membership, future research could explore potential interactions between gender and the school climate predictors. Such research should specifically focus on gender differences and be theoretically informed by previous findings on gender disparities. This approach could offer insights into the interplay between gender dynamics and school environments in influencing adolescent mental health.

While acknowledging its limitations, the study also presents notable strengths. Key among these are the comprehensive multi-informant approach, the inclusion of various school contextual factors and the independent assessment of exposure and outcomes. This design ensures that school climate assessments are separate from health assessments made by younger students, mitigating potential biases in several ways. By having different groups assess the predictors (school climate) and outcomes (health), the risk of inflated associations due to common method bias is minimized. Additionally, separating the sources of assessment reduces the likelihood that the mental health status of younger students will influence their reporting on school climate. Although not a perfect solution for establishing causality, this design introduces temporal separation, helping to mitigate reverse causation concerns. Moreover, using different respondents for school climate and mental health outcomes provides independent validation of the measures, enhancing the robustness of the findings.

In conclusion, this study offers several insights into the association between school climate and the trajectories of adolescents’ depressive symptoms. One key finding is that, despite exploring numerous factors that constitute a substantial portion of the overall ‘school experience’, only a few seem to be relevant for depressive symptoms. The findings highlight the importance of certain factors, particularly those related to the academic and community environment [12,13], in shaping students’ mental health. Specifically, it underscores the significance of interpersonal relationships, including positive leadership from actively supportive principals, and strong teacher expectations for student performance and behaviour, in fostering positive mental health outcomes among youth.

## Supplemental Material

sj-docx-1-sjp-10.1177_14034948241277048 – Supplemental material for Trajectories of depressive symptoms in early to mid-adolescence: associations with school pedagogical and social climate Supplemental material, sj-docx-1-sjp-10.1177_14034948241277048 for Trajectories of depressive symptoms in early to mid-adolescence: associations with school pedagogical and social climate by Cristian Bortes in Scandinavian Journal of Public Health
