# Supplementary material for: Trajectories of depressive symptoms in early to mid-adolescence: associations with school pedagogical and social climate
Source: Scand J Public Health. 2024 Oct 24;53(8):844–53. doi: 10.1177/14034948241277048 (PMC12619849; doi:10.1177/14034948241277048)
Supplement: sj-docx-1-sjp-10.1177_14034948241277048 – Supplemental material for Trajectories of depressive symptoms in early to mid-adolescence: associations with school pedagogical and social climate [file sj-docx-1-sjp-10.1177_14034948241277048.docx]

**Supplementary materials**

**Figure A1:** Flow chart of study participation

**Table A1:** The PESOC instrument for teachers (T-PESOC)

**Table A2:** The PESOC instrument for students (S-PESOC)

**Table A3:** Descriptive statistics and reliability coefficients for the PESOC questionnaires

**Table A4:** Summary of missing data patterns for the variable CES-DC

**Figure A1**: Flow chart of study participation

541 eligible schools

6 schools ineligible

434 schools declined participation

101 schools agreed to participate

12 512 eligible 7^th^ grade students

8 535 7^th^ grade students had no parental consent to participate

3 959 7^th^ grade students with parental consent to participate in the study

*9^th^ grade:*

Individual questionnaires: 3 351

Parental questionnaires: 3 199

T-PESOC questionnaires:
3 617 teachers in 81 schools

S-PESOC questionnaires:
9 732 students in 85 schools

*8^th^ grade:*

Individual questionnaires: 3 502

Parental questionnaires: 3 328

T-PESOC questionnaires:
4 163 teachers in 92 schools

S-PESOC questionnaires:
10 657 students in 93 schools

*7^th^ grade:*

Individual questionnaires: 3 671

Parental questionnaires: 3 644

T-PESOC questionnaires:
4 542 teachers in 101 schools

S-PESOC questionnaires:
11 282 students in 101 schools

**Table A1**: The PESOC instrument for teachers (T-PESOC)

| Item nr. | Subscale | Item |
| --- | --- | --- |
| 1 | TE | Our principal has high demands and expectations for student academic results. |
| 2 | TE | There is a norm among staff in this school that all students have the ability to reach the curriculum goals concerning basic knowledge and skills. |
| 3 | TE | The principal has high demands and expectations for student behavior. |
| 4 | TE | All members of the staff in this school have high expectations for student behavior. |
| 5 | PTA | Teachers in this school apply common principles in regard to social training. |
| 6 | PTA | There is a high degree of unity among teachers regarding the school goals. |
| 7 | PTA | If a decision is made concerning a specific educational practice, even teachers who were critical abide by the decision. |
| 8 | PTA | Teachers are aware that in contact with students they should express the school’s values and norms regarding teaching and upbringing. |
| 9 | PTA | There is a well-expressed norm in this school that teachers commence lessons on time. |
| 10 | PTA | The teachers in this school agree on the school’s rules of conduct. |
| 11 | PTA | There is strong agreement among staff in this school on pedagogical issues. |
| 12 | PTA | If a student requires extra teaching in this school are generally willing to help. |
| 13 | PTA | Th principal, senior management and the teachers agree in regard to the school’s basic educational and social goals. |
| 14 | SF | Social relationships between teachers and students are good in this school. |
| 15 | SF | It is a well-established policy that teachers show respect for students. |
| 16 | SF | It is a well-established policy that students are encouraged to be responsible for their studies. |
| 17 | SF | Students conduct many activities that they initiate on their own. |
| 18 | SF | Teachers on this school have time for students who wish to speak about something other than teaching and learning. |
| 19 | SF | The principle encourages the development of a pleasant school environment for students. |
| 20 | BA | It is well-known in this school that it is the quality of the teaching that is decisive for individual students’ results, not home environment, or intellectual ability. |
| 21 | BA | It is well-known by the staff that knowledge goals are the most important task of this school. |
| 22 | BA | In this school it is well understood that students can learn what is required of then even if they have less favorable home environments for learning. |
| 23 | HOME | It is part of this school’s policy to give parents information on how they can support their children’s schoolwork. |
| 24 | HOME | Parents are always informed if a child behaves badly in school. |
| 25 | HOME | Parents are well informed about this school’s demands and expectations for students. |
| 26 | HOME | The majority of parents participate in parent meetings. |
| 27 | HOME | It is a well-established policy in this school that parents participate in discussions concerning measures for students who risk not reaching knowledge goals. |
| 28 | TC | I have the support of my colleagues in understanding and solving disorder problems in the classroom. |
| 29 | TC | I have the support of my colleagues in selecting teaching content and methods. |
| 30 | TC | If you feel the need to discuss a teaching problem, you can always find a colleague at school who is willing to discuss it with you. |
| 31 | TC | The schools more experienced teachers give active support to the newly examined teachers and to the less experienced teachers. |
| 32 | TPD | The absolute majority of teachers in this school are enthusiastic and committed to their work. |
| 33 | TPD | Most teachers in this school are self-confident when called upon to state their views at conferences and meetings. |
| 34 | TPD | Most teachers in this school are self-confidents when it comes to maintaining discipline and order in the classroom. |
| 35 | TPD | Most of the teachers in this school are confident educators. |
| 36 | TPD | In this school you feel that you can develop as an educator. |
| 37 | TA | It is the ambition of the teachers in this school to practice teaching, which stimulates creative thinking, provides training in problem solving, and is intellectually challenging. |
| 38 | TA | In this school, teachers are able to use the time allocated to teaching for teaching. |
| 39 | TA | In this school student work are displayed on walls. |
| 40 | TA | There is a policy in this school that no student will leave this school without gaining basic knowledge and skills. |
| 41 | TA | In this school all students gain knowledge and skills despite their social or ethnic background. |
| 42 | TA | Students must acquire the basic knowledge and skills before they are allowed to proceed to the next phase of instruction. |
| 43 | EV | Regular evaluation of student results is used as the basic for planning teaching in this school. |
| 44 | EV | In this school evaluation is an important instrument to ensure students reach the knowledge goals. |
| 45 | P | The principal in this school takes the responsibility for applying the nationally established demand levels for teaching and criterion for assessing student knowledge levels. |
| 46 | P | In this school the principal gives strong priority to pedagogical issues compared with administrative tasks and contacts outside school. |
| 47 | P | The principal often shows strength in decision-making. |
| 48 | P | The delegation of teachers to classes is steered predominantly by pedagogy. |
| 49 | P | The principal makes a point of being visible in the school. |
| 50 | P | In service training and study days are carefully planned and aim at specific pedagogical issues. |
| 51 | P | The principal is accessible if needed to solve conflicts between teachers. |
| 52 | P | In this school the principal gives priority to educational issues in his or her contacts with teachers, students, and parents. |
| 53 | P | The principal in this school is available to discuss teaching issues with teachers. |
| 54 | P | The principal actively participates in teachers’ professional development. |
| 55 | P | My principal is accessible to discuss teaching methods. |
| 56 | P | My principal gives me regular feedback regarding my work in the classroom. |
| 57 | SM | There is always a member of school management accessible to discuss and give advice on issues regarding teaching and education. |
| 58 | SM | There is always a member of school management accessible to discuss and give advice on issues regarding order in the classroom. |
| 59 | SM | There is always a member of school management accessible to discuss and give advice on how to solve conflicts with students. |

*Note:* TE = Teachers expectations for students’ behavior and academic performance; PTA = Perceived teacher agreement about school goals, norms, and rules; SR = Social rules regarding students’ behavior and achievement; SF = Student focus; BA = Basic assumptions about students’ ability to learn; HOME = Communication between school and home; TC = Teacher interaction and cooperation; TWE = Teachers work environment; TA = Teaching activities; EV = Evaluation of students’ academic progress; P = Principal’s pedagogical leadership; SM = Teachers’ perception of school managements’ involvement and support

**Table A2**: The PESOC instrument for students (S-PESOC)

| Item nr. | Subscale | Item |
| --- | --- | --- |
| 1 | E | I feel that almost all my teachers believe that I can pass and get good grades if I want to |
| 2 | E | In this school, teachers expect students to pass all subjects |
| 3 | E | All my teachers believe I can get accepted into gymnasium (three years theoretical or vocational track) |
| 4 | E | My teachers think that classes should be peaceful and quiet. |
| 5 | P | Almost all my teachers make us work hard on our school assignments |
| 6 | P | My teachers seem to like their job |
| 7 | P | The teachers at this school seem to consider teaching to be important |
| 8 | P | In this school all teachers seem to care for the students |
| 9 | P | The teachers are always on time for class |
| 10 | P | The teachers seem to consider every minute of class to be important |
| 11 | P | All teachers apply the same rules of conduct |
| 12 | P | The teachers make sure that the classroom is peaceful and quite |
| 13 | P | The teachers in this school respect the students |
| 14 | P | The atmosphere among teachers sems good |
| 15 | P | All my teachers are interested in me as a person |
| 16 | P | This school is ok |
| 17 | TS | If you start to study more, the teachers immediately notice |
| 18 | TS | If there is something you do not understand you can be almost certain that the teacher will help you during class |
| 19 | TS | I can ask my teacher for help outside the classroom, if there is something I do not understand during class, or when doing my homework |
| 20 | TS | I usually have enough time to complete all my tasks during class |
| 21 | TS | I can speak with my teacher about matters not related to school |
| 22 | TA | Almost all my teachers teach interesting classes |
| 23 | TA | In most classes, teachers ask questions that require you to think for yourself in order to answer |
| 24 | TA | Most of the time it is fun learning new things in school |
| 25 | TA | The classes at my school make me understand more and more about society and how things are related |
| 26 | TA | After going over something, our teacher will give us an assignment to work on individually or in a group |
| 27 | TA | Teachers often explain why it is important to learn something |
| 28 | TA | In almost all subjects we get to search for information by ourselves and write our own work |
| 29 | TA | My teachers let all students answer the questions |
| 30 | TA | If I do well, my teachers praise me |
| 31 | TA | I more often receive encouragement and praise than reprimands |
| 32 | TA | The teachers require us to work on assignments throughout class |
| 33 | TA | When working on individual assignments, I always know what it is I am supposed to do |
| 34 | TA | If there is a risk that you will fail the knowledge goals, you will receive extra tutoring |
| 35 | TA | I receive information about what I need to do to get certain grades |
| 36 | TA | When working in groups, we all understand what we have to do |
| 37 | SP | The students at this school often organize activities such as dance, running a café, stage plays, etc. |
| 38 | SP | We students take part in discussions about matters like rules of conduct and the school environment |
| 39 | SP | We students are allowed to take part in preparing classes |
| 40 | SE | We students have access to good facilities during breaks and free periods |
| 41 | SE | The food in school is good |
| 42 | SE | The environment at the school is nice |
| 43 | SH | Teachers often contact my parents to let them know how I am doing in school |
| 44 | SH | If I was to get into a fight or behave badly, my parents would soon be notified |
| 45 | SH | At parent-teacher conferences, my parents are informed about my developments |
| 46 | SH | At parent-teachers conferences, my parents and I are told what I can do to get certain grades (A-F). |
| 47 | SH | My parents are often provided with information about what is going on in school. |
| 48 | SM | Our principal and/or assisting principal seem very interested in what we learn in school |
| 49 | SM | Our principal and/or assisting principal seem very interested in classes being peaceful and structured |
| 50 | SM | I see our principal or assisting principal in our school almost every day |
| 51 | SM | Our assisting principal recognizes students at our school |
| 52 | SM | Someone from the school management or other teachers usually visit our classes to see what we are doing |
| 53 | SM | Our principal seems interested in us students |

*Note*: E = Expectations; P = Perception of teacher norms; TS = Teacher support; TA = Teaching activities; SP = Student participation; SE = School environment; SH = School and home; SM = School management

**Table A3:** Descriptive statistics and reliability coefficients for the subscales of the PESOC Questionnaires

| PESOC subscale | Range | Mean (std deviation) | Cronbach’s  alpha |
| --- | --- | --- | --- |
| **Teacher PESOC** |  |  |  |
| Total teacher PESOC | 2.49-3.73 | 3.24 (0.19) |  |
| Basic assumptions about students’ ability to learn | 2.90-3.85 | 3.47 (0.15) | 0.61 |
| Evaluation of students’ academic progress | 2.39-3.81 | 3.14 (0.24) | 0.78 |
| Teachers’ expectations for students’ behavior and academic performance | 2.90-3.88 | 3.51 (0.19) | 0.62 |
| Communication between school and home | 2.57-3.80 | 3.40 (0.17) | 0.72 |
| Perceived teacher agreement about school goals, norms, and rules | 2.72-3.78 | 3.30 (0.19) | 0.82 |
| Principal’s pedagogic leadership | 1.85-3.86 | 2.95 (0.38) | 0.90 |
| Student focus | 2.99-3.79 | 3.47 (0.14) | 0.67 |
| Teachers’ perception of school managements’ involvement and support | 1.81-4.00 | 3.07 (0.43) | 0.91 |
| Teaching activities | 2.71-3.74 | 3.26 (0.20) | 0.67 |
| Teacher interaction and cooperation | 2.88-3.86 | 3.46 (0.19) | 0.79 |
| Teachers’ confidence and professional development | 2.69-3.84 | 3.37 (0.20) | 0.77 |
| **Student PESOC** |  |  |  |
| Total student PESOC | 2.48-3.48 | 2.97 (0.15) |  |
| Expectations | 3.16-3.81 | 3.53 (0.10) | 0.42 |
| Perception of teacher norms | 2.47-3.61 | 3.10 (0.17) | 0.80 |
| School environment | 1.87-3.65 | 2.70 (0.31) | 0.51 |
| School and home | 2.74-3.67 | 3.20 (0.17) | 0.67 |
| School management | 1.58-3.61 | 2.51 (0.38) | 0.77 |
| Student participation | 1.78-3.32 | 2.47 (0.24) | 0.53 |
| Teaching activities | 2.55-3.41 | 2.95 (0.14) | 0.82 |
| Teachers’ support | 2.52-3.51 | 3.06 (0.16) | 0.60 |

**Table A4:** Summary of missing data patterns for the variable CES-DC across three time points

| **Pattern** | **CES-DC t1** | **CES-DC t2** | **CES-DC t3** | **Frequency** |
| --- | --- | --- | --- | --- |
| 1 | x | x | x | 2714 |
| 2 | x | x |  | 350 |
| 3 | x |  | x | 229 |
| 4 | x |  |  | 166 |
| 5 |  | x | x | 257 |
| 6 |  | x |  | 62 |
| 7 |  |  | x | 59 |

x = not missing

Total frequency = 2714 + 350 + 229 + 166 + 257 + 62 + 59 = 3837

**Pattern 1**: Complete data for all three variables (2714 participants)
**Pattern 2**: Missing CES-DC t3 (350 participants)
**Pattern 3**: Missing CES-DC t2 (229 participants)
**Pattern 4**: Missing CES-DC t1 (166 participants)
**Pattern 5**: Missing CES-DC t2 and CES-DC t3 (257 participants)
**Pattern 6**: Missing CES-DC t1 and CES-DC t3 (62 participants)
**Pattern 7**: Missing CES-DC t1 and CES-DC t2 (59 participants)
